# Comparison of Measurements for Recording Postural Control in Standing and Seated Position in Healthy Individuals

**DOI:** 10.3390/jfmk9040178

**Published:** 2024-09-27

**Authors:** Philipp Floessel, Franziska Hammerschmidt, Jan Jens Koltermann, Justin Foerster, Heidrun Beck, Alexander Carl Disch, Thomas Datzmann

**Affiliations:** 1 University Center for Orthopedics, Trauma & Plastic Surgery, Faculty of Medicine Carl Gustav Carus, Technische Universität Dresden, Fetscherstrasse 74, 01307 Dresden, Germany; franziska.hammerschmidt@uniklinikum-dresden.de (F.H.); justin.foerster@uniklinikum-dresden.de (J.F.); heidrun.beck@ukdd.de (H.B.); alexander.disch@ukdd.de (A.C.D.); 2 Consulting Engineer for Metrology and Data Science, Bahnhofstraße 33, 03046 Cottbus, Germany; koltermann.jan@gmx.de; 3 Medizinische Fakultät Carl Gustav Carus, Center for Evidence-Based Healthcare, TU Dresden, Fetscherstrasse 74, 01307 Dresden, Germany; thomas.datzmann@ukdd.de

**Keywords:** postural control sitting position, standing balance, center of pressure, neuromuscular control

## Abstract

**Background**: A standard method of assessing postural control is to measure while standing. However, its implementation is usually limited. Recording postural control directly on the trunk in a seated position could provide an alternative diagnostic method for quantifying neuromuscular control. **Methods**: A comparison of center of pressure (CoP) measurements in the standing and sitting positions was performed on 66 healthy adult subjects. The reliability of the measurements in the sitting position was tested in 23 subjects. In addition, the extension force of all test subjects was recorded. **Results**: The assessments of CoP fluctuations in standing and seated positions showed adequate agreement (deviation 9.1%). Furthermore, good internal consistencies with a sufficient test–retest reliability could be demonstrated for the measurements in seated position. Both CoP measurement methods showed a comparable Spearman correlation to obtained extension force measurements (standing: 0.24, seated: 0.23). **Conclusions**: Our results show that recording CoP fluctuations in the sitting position is a reliable and valid adjunct to single-leg stance measurements. It could serve as an additional alternative to quantify neuromuscular control in impaired patients who cannot adequately perform the single-leg stance. In addition, measurement in the seated position allows direct recording of neuromuscular control at the trunk.

## 1. Introduction

Maintaining an upright posture and achieving balance during movement necessitates the coordinated engagement of various muscle groups and their neuromuscular control [1]. In this context, the muscles of the lower extremities and the trunk are particularly significant [2,3].

In various pathologies such as Parkinson’s disease, multiple sclerosis, and obesity, both muscle tone and the efficient activation of the aforementioned muscle groups are compromised [4,5]. This impairment leads to reduced postural control, which is associated with an increased risk of falls [4,6]. Additionally, the occurrence of chronic back pain has been described at a physiological level, characterized by a lack of neuromuscular control of the muscles surrounding the trunk [7,8]. Reduced postural control is also observed in patients with low back pain [9].

Given the significant cost to the healthcare system of falls and loss of work capacity due to back pain, early identification of patients at risk is of paramount importance [10].

A common assessment to determine the level of postural control involves measuring postural control while standing on a force plate [11,12]. The center of pressure (CoP), defined as “the point at which the pressure of the body over the soles of the feet would be if it were concentrated at one point”, can be extracted as a measurement variable. The distance traveled by the CoP during a measurement provides insights into balance control and neuromuscular control [13].

However, the implementation of this diagnostic method is not fully applicable in all patient settings. For example, the literature suggests that patients with multiple sclerosis or Parkinson's disease may be at increased risk of falls [4,14]. Assessing postural control directly on the trunk in a seated position could serve as an alternative diagnostic method for quantifying neuromuscular control, potentially meeting the need for a safe routine test in clinical settings [15,16].

In addition, measuring CoP in the seated position may provide a more sensitive way of quantifying the specific trunk strength contribution to overall postural control, as CoP in the standing position is significantly influenced by lower limb strength. The aim of this study was to compare measures of postural control in the single-legged standing position with those in the seated position in relation to back extension strength. The potential benefit would be to provide a safe alternative measurement method for assessing postural control in clinical patient settings and to more sensitively diagnose individual sub-components related to the trunk and lower extremities in relation to overall postural control.

**Figure 1:**
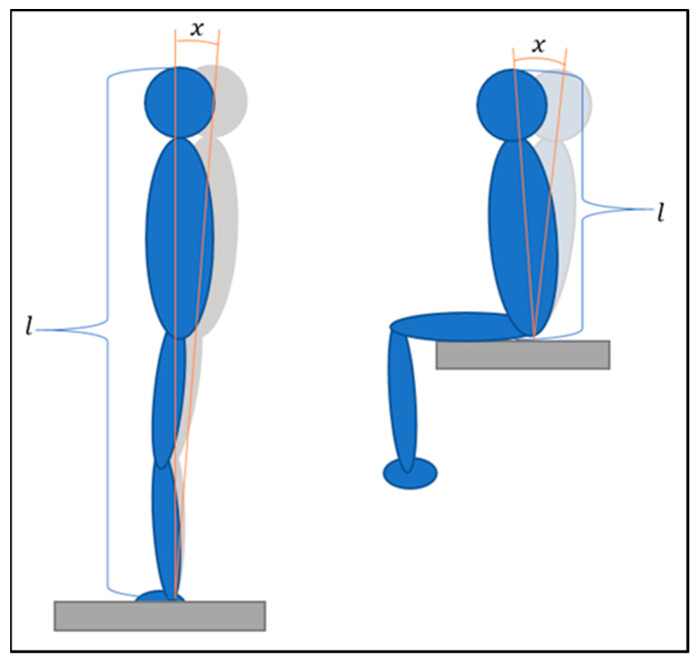
Angle of the upper body over the force plate during standing (**left**) and sitting (**right**) position.

The center of pressure (CoP) was measured using a force plate, and back extension strength was measured using a corresponding power machine. From a biomechanical perspective, the two assessments—sitting and standing—are comparable. Essentially, both variants of the CoP trajectory can be considered as a single inverted pendulum. The difference in trajectory length between the variants represents a linear relationship. For approximately the same pendulum angle of the upper body, the shorter pendulum results in a shorter path over the force plate. Due to the larger support surface in the seated position, it can be assumed that the angle of deflection at the hip joint is not greater than the angle of deflection at the ankle joint in the standing position [17,18]. Refer to Figure 1 for further illustration.

The gold standard systems for recording the center of pressure (CoP), due to their measurement accuracy, are the force plates manufactured by Kistler and AMTI [19,20]. In this context, an alternative, much lighter, and more compact measurement plate—a modified Wii^®^ balance board—was used in the current study. The modified Wii^®^ Balance Board is a modification according to Koltermann et al. 2017 [21]. The balance board meets the requirements for valid and reliable recording of the CoP trajectory in a standing position and can also be used portably, allowing for measurements to be taken in an elevated position to record postural control in a seated position [21,22].

## 2. Materials and Methods

### 2.1. Participants

In the cross-sectional study, 66 subjects (34 female) between 19 and 58 years of age participated. A necessary sample size of 54 participants was calculated in advance. Assuming a power of 95% with a 5% significance level and a permissible deviation of 1.5 times the expected standard deviation of the measurement methods, a minimum case number of 54 patients was set in advance [23], determined with the software MedCalc from MedCalc Software version 20.027 Ltd. (Ostend, Belgium) [24]. In principle, we would expect five percent background noise with this method after all, assuming that the error is normally distributed. Therefore, the authors specified in advance that a deviation of up to 10% could be tolerated for the measurements to be considered consistent [23].

The study received ethics approval, and all participants gave their written informed consent. All examinations were carried out in a center certified by the German Olympic Sports Confederation. Volunteers were included only if they answered “no” to all questions of the “Entry Questionnaire for Health Risk Assessment for Athletes” of the German Society for Sports Medicine and Prevention [25]:

Have you ever been told by a doctor that you have a cardiac condition and that you should only engage in exercise or sports under medical supervision?
Have you experienced chest pain in the past month, either at rest or during physical exertion? Do you have difficulty breathing at rest or during physical activity? Have you ever fallen due to dizziness or lost consciousness? Do you have any bone or joint problems that could be exacerbated by physical activity? Have you ever been prescribed medication by a doctor for high blood pressure, or for a heart or respiratory condition?
Are you aware of any other reason why you should not engage in physical activity?

Had no lower extremity injuries in the last 24 months and reported neither acute nor other back pain within the last three months? (PAR-Q-Fragebogen, DGSP) [25]. Back pain was queried using Korff’s Chronic Pain Grade Questionnaire [26]. Additional inclusion criteria were that subjects had to be between 18 and 60 years of age and able to stand on one leg. Exclusion criteria were individuals with an endoprosthesis of the lower extremities, individuals suffering from vertigo, and pregnant individuals.

### 2.2. Equipment

Postural control was measured using a balance board, which is a technical advancement of a Nintendo Wii^®^ balance board. The Wii^®^ balance board is mechanically loadable up to 150 kg, with the sensors delivering accurate measurements up to 120 kg when using the specified amplifier. Operation up to 110 kg poses no issues for this setup. The Wii^®^ balance board is equipped with four strain gauge sensors, each configured as a full-bridge measurement. The analog signal processing includes a differential amplifier and an output amplifier stage. Calibration and validation of the conversion were performed as described by Koltermann et al. 2017. The sampling rate was set at 1 kHz [21].

For converting the analog measurement data into a digital format, a National Instruments NI USB 6001 converter with a 14-bit sampling rate was used, as recommended by Koltermann et al. 2022 [27]. On the software side, raw data were recorded using LabVIEW 2014 from National Instruments (Austin, TX, USA). The processing of the measured data from the balance board was also conducted using LabVIEW 2014. Prior to calculating the CoP trajectory, the raw data were filtered using a Butterworth 3rd-order low-pass filter. For the entire cohort, a filter was applied according to the procedure described in the literature, which determined the cutoff frequency [28]. Subsequently, the CoP trajectories were determined, and CoP fluctuation was calculated across the entire transverse plane. Extension force was recorded concentrically using a dynamometer (Ferstl GmbH, Hemau, Germany).

### 2.3. Procedures

The subjects’ CoP, recorded as the distance traveled by the center of pressure during the measurement in centimeters, was measured in a standardized order in two different conditions. First, four trials were performed while standing on one leg, followed by two trials while sitting, both with eyes open.

Before each subject run, the balance board underwent an internal calibration loop.

The measurements were taken in a standing position with one leg. It can be assumed that a greater neuromuscular effort is required to maintain postural control in the one-legged stance compared to the two-legged stance [29,30]. This should allow for better comparability with the seated measurements and the trunk strength measurements in the statistical analysis. Although the lower extremity has a significant influence on the CoP deflection in the standing measurements, there is still comparability between the two measurement methods in the study [31,32,33]. However, a direct comparison between the two is neither feasible nor recommended and is not part of the study.

The order of monopedal stances (left leg, right leg) was randomized. The measuring time per condition was 60 s. There was a rest period of one minute between each measurement. Standardization was used to avoid any fatigue effects due to decreasing concentration or strength. Any signs of increasing instability or loss of balance before the end of this time were documented, and the results were corrected by hand in addition to automatic scoring.

**Figure 2:**
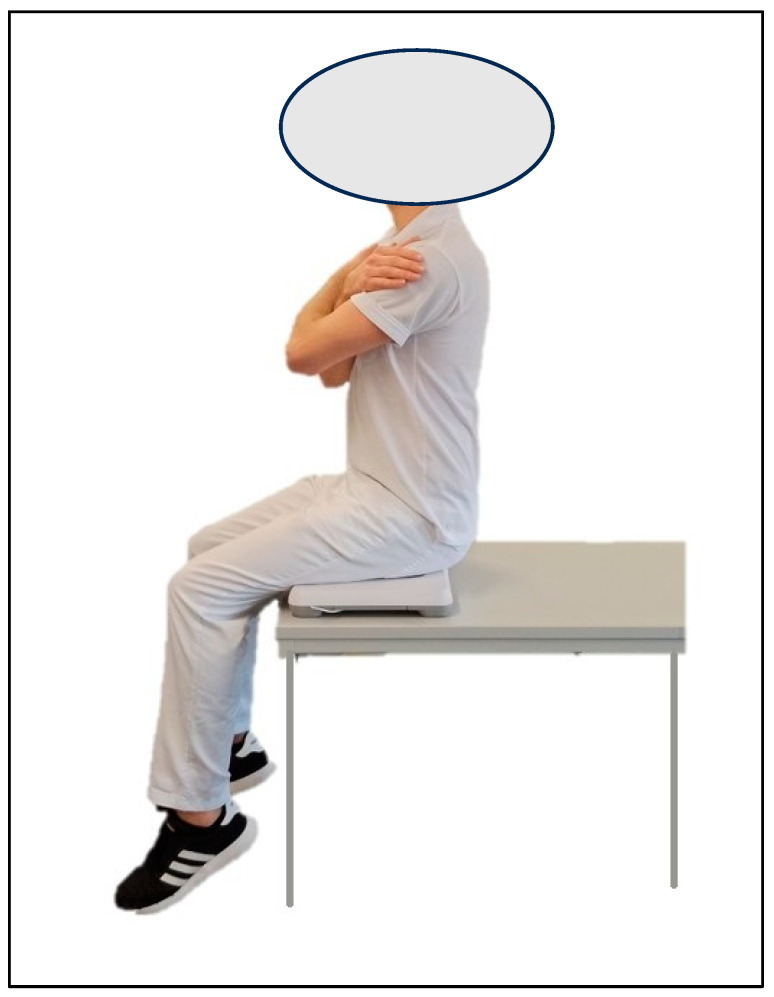
Test person in seated position on the balance board with an active upright upper body positioning during the measurements.

The stance position was standardized by optical markings on the force plate indicating the position of the foot. During the measurements, the hands were placed at the sides of the standing position, and the lifted leg was not allowed to rest on the standing leg. A visual marker was placed four meters from the measurement position at a height of 1.7 m. Subjects were asked to visually fixate this point during the measurements. All measurements were performed barefoot. For the seated assessments, the subjects sat in an active upright position centered on the balance board. They were also asked to look at a marker four meters away. During the trials, the subjects crossed their hands in front of their chests. The legs had to remain parallel. The height of the test position was adjustable so that the subjects’ legs hung freely 50 cm above the floor (Figure 2).

Back extension strength was measured over ten isokinetic repetitions performed in a seated position. A total of two tests were performed with a 60 s rest interval between them. The posture of each participant was automatically calibrated using the appropriate program (IsoMed 2000 Back Module from D&R Ferstl GmbH), ensuring that the back was firmly positioned against the cushion and the feet were in contact with the floor plate. The zero point of the upper body was set at 105°, and the measurement speed was set at 60°/s as a baseline configuration. A belt was also placed around the abdomen.

### 2.4. Statistical Analysis

The autocorrection checks the CoP records for illogical extremes caused by brief losses of control. These are smoothed by a partial moving average. If the operator has left the plate during the measurement, the measurements are sorted out manually. After automatic correction and manual correction, the distance covered by the CoP during the one-minute measurement was obtained in centimeters. The agreement between the measurement of the CoP fluctuation in the standing and sitting positions on the balance board was assessed using mean difference plots and Spearman correlation coefficients [34]. Therefore, the measurements of the CoP could be directly compared without any further transformation. 

A Spearman correlation coefficient was obtained to compare the CoP fluctuation values (a) in the standing position and (b) in the sitting position. A correlation analysis was also performed between the standing and sitting measurements and the corresponding distension force measurements in Nm/kg. We also assessed whether the CoP values obtained in the seated position could be used to discriminate between subjects with low and high values of extension force. We used a mean split of the extension force values (mean 3.32 Nm/kg) and compared the mean values of the CoP fluctuation using the Welch *t*-test.

Reproducibility of individual measurements was tested using a two-tailed *t*-test (TOST) on the individual, raw CoP fluctuation values with an allowed discrepancy of 1 times the expected standard deviation (epsilon) and an alpha error of 10% [35]. Equivalence was tested using the package “EQUIVNONINF” [36] for the statistical software R version 4.1.0 [37,38]. Of 66 participants, 23 subjects were measured twice at two time points T0 and T1, about 7 days apart, in seated position on the balance board.

The back extension force was recorded using an appropriate software program. The maximum torque (in Newton-meters), average torque (in Newton-meters), and total work (in joules) were determined automatically. Extension power was then calculated based on the average torque and the subject’s body weight.

## 3. Results

After reviewing the inclusion and exclusion criteria, 66 healthy subjects were included in the study. The mean age of the participants was 28.59 years (SD 7.8). They were relatively young, with an age distribution ranging from 19 to 58 years, and had a normal cross-sectional mean weight, but with a rather heterogeneous distribution of their BMI ranging from 15.7 kg/m^2^ to 43.1 kg/m^2^.

In general, the CoP fluctuation in standing position is higher than the CoP fluctuation in seated position (Table 1), which is consistent with previous study results [21,39]. 

When looking at the individual values in standing and sitting, it is noticeable that the ratio of the mean CoP fluctuation is approximately 2:1 (*p* < 0.001, *t*-test). A similar observation was already made by Roerdink et al. in the context of their evaluation [16].

### 3.1. Comparison of Standing and Seated Position on the Balance Board

**Figure 3:**
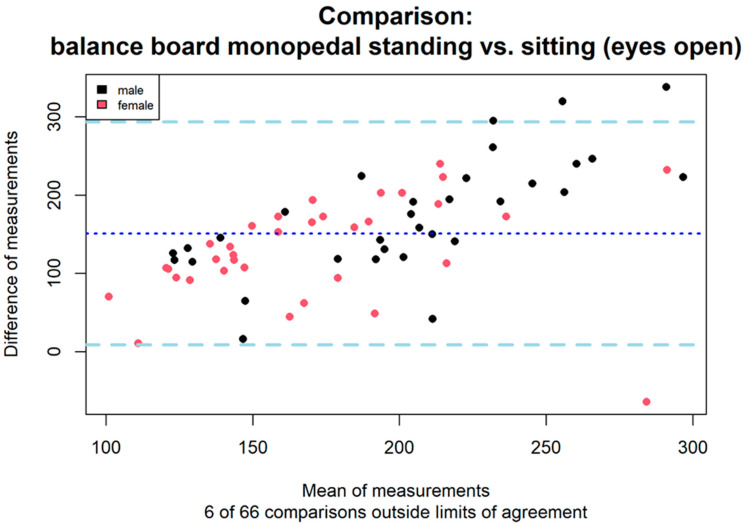
Mean difference plot showing agreement between measurements for CoP in monopedal standing and seated position with eyes open. The thick dashed lines at the top and bottom of the figure represent the limits of agreement, the dotted line represent the mean value. Axis (x, y) units are in centimeters (cm). The x axis shows the mean versus the difference of the two measurements on the y axis. Six of sixty-six comparisons all outside or intersect with the limits of agreement, which corresponds to a deviation of 9.1%. Women and men are color coded for representation purposes only.

Mean difference plots were constructed to compare measurements taken in the standing and sitting positions on the balance board. The mean difference plots showed good agreement between the two instruments. The deviation between the standing and sitting measurements was 9.1% (Figure 3). This is below our pre-determined acceptable deviation of ten percent, so the standing and sitting measurements were in good agreement. Furthermore, the CoP fluctuation measurements in (a) standing and (b) sitting positions had a positive Spearman correlation coefficient of 0.47. Both methods of measuring CoP fluctuation showed a comparable Spearman correlation to the additionally obtained extension force measurements in Nm/kg (a: 0.24, b: 0.23). Although there was a low correlation between CoP fluctuation in the seated position and strength, this was also true for CoP fluctuation values in the standing position (standard measurement). Subjects with low or high extension force values (split by mean extension force value at 3.32 Nm/kg) measured in the sitting position showed a significant difference in mean values (Welch *t*-test—mean1: 100.8 cm, sd1: 31.5 cm, mean2: 122.2 cm, sd2: 49.1 cm, t = −2.06 (95%-CI −42.3/−0.6), *p* = 0.044).

### 3.2. Reliability of the Measurements with the Balance Board

**Figure 4:**
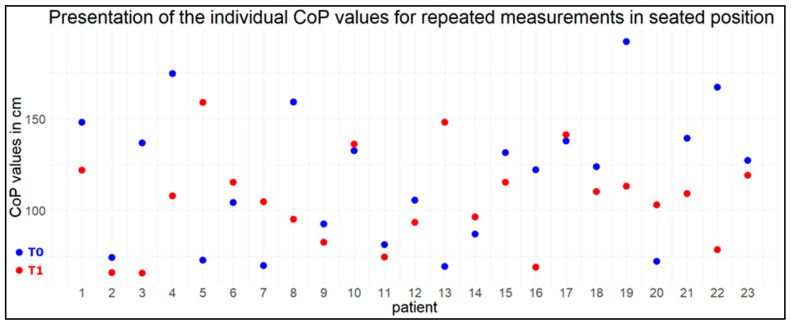
Blue dots represent individual measurements of CoP fluctuations for the first time point T0, red dots the measurements for the same individuals to time point T1.

The equivalence of the individual measurements was demonstrated by the TOST test on the CoP fluctuation values for subjects in the sitting position (equivalence bound 58 cm, mean of the differences: 12.87 cm, *p* < 0.001). The null hypothesis that there was an effect large enough to be of interest was rejected. Therefore, there is no reason to believe that the measurements at the two time points are significantly different (see also Figure 4).

## 4. Discussion

This study compared measurements of CoP fluctuation in the standing and sitting positions on the balance board in healthy adults. Despite the additional problem of stabilization in the frontal plane in the one-legged stance measurement, we were able to demonstrate good agreement between the two measurement methods using mean difference plots. Although different back extension forces act in the sitting and standing positions. The mean difference analysis showed a tolerated deviation of 9.1%. Subjects with low CoP fluctuation values in the standing position also had low CoP fluctuation values measured in the sitting position, and vice versa for high values. However, the Spearman correlation between the two methods was only moderate (correlation coefficient 0.47). Furthermore, the correlation of CoP fluctuation measurements from both methods was comparable in direction and magnitude to that of the stretch force measurements. CoP fluctuation measurements obtained in the seated position showed a significant difference in mean values between subjects with low and high extension forces. 

The clinical added value of the current study is underlined by the fact that the assessment of postural control in a seated position serves as an alternative measurement method compared to diagnostics performed in a standing position. This approach allows for the assessment of superior postural control in individuals with physical limitations in a safe, low-risk environment. Furthermore, the results of this study can be used as a benchmark to more accurately quantify performance deficits in neuromuscular structures in patient populations with low back pain or lower limb injuries, provided the same test design (center of pressure assessment in both standing and sitting positions) is used. In these patient groups, discrepancies between the two diagnostic methods (recording of head trajectories in standing and sitting positions) are expected, depending on the specific limitations encountered. Preliminary studies supporting these conclusions include those by Vuillerme et al. (2004), Barbado et al. (2016), and Roerdink et al. (2011), which demonstrated that sport-specific profiles of postural control can be distinguished between standing and sitting positions [15,16,38]. With this in mind, targeted assessment in a seated position to diagnose trunk stabilizing muscles, alongside measurement of postural control in a standing position to assess specific muscle activity in the lower extremities, appears to be beneficial for certain co-habitants. The results may inform differentiated intervention strategies, allowing for more deficit-oriented training of overall postural control. This approach seems feasible both in clinical settings and for athletes.

In addition to its application in clinical settings, the partial differentiation of neuromuscular control between the trunk and the lower extremities appears to be relevant for optimizing athletic performance and preventing injury. The coordination of the pelvis, the joints, and especially the dynamic stability of the trunk, the pelvis, and the legs is a critical factor for performance, especially in athletes [39,40,41]. Current literature indicates that enhanced trunk stability is associated with a reduced risk of injury and improved athletic performance [42,43,44]. Assessment of neuromuscular control in the seated position may facilitate early identification of weaknesses in trunk control. Further research is needed to determine whether athletes within the same sport, both with and without low back pain, have increased CoP values in the seated position, similar to the findings of Lemos et al. (2010) on postural control assessments in the standing position, and to investigate the influence of extension force on CoP trajectories [12]. It is plausible that assessing postural control in the seated position will provide a more sensitive method of assessing neuromuscular trunk control, allowing earlier identification of patients at risk of pain in elite sport and facilitating the implementation of preventative training interventions. This approach is also applicable to rehabilitation following conditions such as herniated discs. By using combined standing and sitting diagnostics, it may be possible to diagnose neuromuscular control adaptations on a segment-specific basis throughout the course of training therapy, thereby allowing for tailored adjustments to rehabilitation programs. In order to provide a reliable diagnosis of neuromuscular control of the trunk muscles, future studies should investigate the activation of trunk-spanning muscles using electromyographic assessment.

Furthermore, the implementation of the seated measurement should be critically examined. In addition, the demands of the seated method could be increased by having subjects perform the measurements on an unstable surface, such as a hemisphere, to more clearly identify deficits in neuromuscular control. In addition, future work should investigate the effects of targeted core stability programs on measures of postural control in the seated position.

### Limitations

Possible limitations of the measurement setup could be that the subjects have positioned themselves too far to the front or rear of the measurement plate, and therefore the center of gravity of the body is not centered. This could lead to limited recording and falsification of the CoP variations. It is also conceivable that the different thigh lengths of the subjects could result in different leverage effects. For example, long thighs could act as a compensating support for the body's center of gravity, so that a calmer sitting position and thus a lower CoP fluctuation value might not be due to good trunk stability, but rather to better leverage conditions.

It is also unclear what effect active control of the transversus abdominis muscle has on the resulting CoP fluctuation value. All subjects were instructed to sit upright under control of the transverse abdominis muscle, but this could not be verified. This could also affect the CoP fluctuation value. In addition, the current measurement setup does not allow verification of whether the subjects gained upper body stability primarily by adopting a passive sitting posture. In this case, the increased load on the capsular and ligamentous structures would lead to a reduction in active neuromuscular holding work. It may be possible to quantify the proportion between passive and active stabilization work by evaluating different CoP frequency spectra [43]. Further work on this is necessary.

## 5. Conclusions

Our analysis shows that postural control measured in standing and seated position with the balance board is in good agreement with each other. Consequently, the assessment of neuromuscular control in a seated position could represent an additional method for the quantification of neuromuscular control of the trunk, which is not influenced by the lower extremities. This method can be employed for a diverse range of patients who are unable to be measured in a standing position due to impairments. It may also provide a more accurate measure of trunk stability or the results of trunk-specific interventions. These assumptions need to be confirmed in future studies with different patient groups.

## Figures and Tables

**Table 1:** Distance covered by the CoP during one measurement (60 s) in different positions and extension force.

	Mean	±SD
CoP balance board monopedal standing (average value of the two limbs) in cm	256.71	5.14
CoP balance board sitting (average value) in cm	124.06	20.12
Extension force in Nm/kg	3.32	0.798

## Data Availability

The original contributions presented in the study are included in the article, further inquiries can be directed to the corresponding author.
